# Biodiversity's Big Wet Secret: The Global Distribution of Marine Biological Records Reveals Chronic Under-Exploration of the Deep Pelagic Ocean

**DOI:** 10.1371/journal.pone.0010223

**Published:** 2010-08-02

**Authors:** Thomas J. Webb, Edward Vanden Berghe, Ron O'Dor

**Affiliations:** 1 Department of Animal and Plant Sciences, University of Sheffield, Sheffield, United Kingdom; 2 Ocean Biogeographic Information System, Institute of Marine and Coastal Sciences, Rutgers University, New Brunswick, New Jersey, United States of America; 3 Census of Marine Life, Consortium for Ocean Leadership, Washington, D. C., United States of America; NatureServe, United States of America

## Abstract

**Background:**

Understanding the distribution of marine biodiversity is a crucial first step towards the effective and sustainable management of marine ecosystems. Recent efforts to collate location records from marine surveys enable us to assemble a global picture of recorded marine biodiversity. They also effectively highlight gaps in our knowledge of particular marine regions. In particular, the deep pelagic ocean – the largest biome on Earth – is chronically under-represented in global databases of marine biodiversity.

**Methodology/Principal Findings:**

We use data from the Ocean Biogeographic Information System to plot the position in the water column of *ca* 7 million records of marine species occurrences. Records from relatively shallow waters dominate this global picture of recorded marine biodiversity. In addition, standardising the number of records from regions of the ocean differing in depth reveals that regardless of ocean depth, most records come either from surface waters or the sea bed. Midwater biodiversity is drastically under-represented.

**Conclusions/Significance:**

The deep pelagic ocean is the largest habitat by volume on Earth, yet it remains biodiversity's big wet secret, as it is hugely under-represented in global databases of marine biological records. Given both its value in the provision of a range of ecosystem services, and its vulnerability to threats including overfishing and climate change, there is a pressing need to increase our knowledge of Earth's largest ecosystem.

## Introduction

The tragedy of studying biodiversity during an extinction crisis is that we are losing our subject matter faster than we are able to describe it [1]. This is especially true in the marine environment, where the need to value and conserve taxa and habitats that we know little about has been termed a paradox of marine conservation [2]. Recent efforts by international networks such as the Marine Biodiversity and Ecosystem Functioning EU Network of Excellence (www.marbef.org) and the Census of Marine Life (www.coml.org) have substantially advanced our knowledge of the marine diversity of specific regions [3], [4] and habitats [5], in large part by harnessing the power of integrated databases [6]. As well as highlighting what we know about marine biodiversity, however, such databases also allow us to quantify what we do not know. For instance, global synthetic analyses have revealed that even for the best known marine taxa, regional inventories remain worryingly incomplete [7]. Spatial biases are also apparent. In particular, the deep pelagic ocean is revealed as biodiversity's big wet secret.

The marine pelagic environment is the open oceans and seas, away from the coasts and above the sea bed; and the deep pelagic ocean is typically defined as that part of the water column deeper than 200m. It constitutes a vast biovolume of space in which organisms can exist – by far the largest on Earth at over a billion km^3^ [8]-[11]. We know that this vast realm and the organisms living in it provide globally important ecosystem services [11], including the support of fisheries, the provision of a range of natural products of potential use in medicine and other applications, as well as the regulation of climate and ocean chemistry through the capture and storage of atmospheric carbon and the production of marine carbonate. But, the limits of our knowledge of this system are continually exposed by the regular discovery of new clades of often large, active and conspicuous organisms [12] whenever surveys are undertaken. Even a charismatic, widely distributed and very large species, the megamouth shark *Megachasma pelagios*, was not discovered until 1976, and has since been recorded so rarely that each individual specimen has become well known [13].

Although it is generally recognised that our knowledge of the deep pelagic ocean is inadequate [9], [11], it is useful to understand the extent to which this is unusual compared with other regions of the marine environment. Here, we address this question using the most extensive compilation in existence of the spatial distribution of marine taxa. We estimate the location in the water column of c.7 million georeferenced records of marine organisms recorded in the Ocean Biogeographic Information System to provide a graphical summary of the three dimensional distribution of recorded global marine biodiversity.

## Methods

### The Ocean Biogeographic Information System

The objective of OBIS, the Ocean Biogeographic Information System, is to mobilize, integrate and quality control raw biogeography data from many different sources, and to make the resulting data compilation freely and openly available through its international portal (http://www.iobis.org) and other suitable channels [14]. OBIS was created as the data integration component of the Census of Marine Life [15], [16], but also holds data originating from its 14 Regional OBIS Nodes, from Thematic Nodes such as FishBase, ICoMM or OBIS SEAMAP, and from independent providers. At present, OBIS makes available through its portal data from well over 700 individual data sets, and has more than 22 million records (a species at a location). It is the largest primary provider of marine biogeographical information, and one of the main providers of data to the Global Biodiversity Information Facility (GBIF). In June 2009, OBIS was adopted by the Intergovernmental Oceanographic Commission as one of its activities under its International Oceanographic Data and Information Exchange programme. Although there are known issues in OBIS with regards to taxonomic completeness, geographical biases, and biogeographic accuracy [7], [17], it remains the most complete and comprehensive data repository in existence on the distribution of marine taxa.

### Data extraction and processing

We extracted all records from OBIS for which sample depth (m) was recorded as well as the latitude and longitude of the sampling event. We used the sample latitude and longitude to determine the depth of the sea floor (bottom depth, m) at that location using ETOPO1 [18]. ETOPO1 is a 1 arc-minute global relief model of Earth's surface including ocean bathymetry, built from numerous global and regional data sets, and available from http://www.ngdc.noaa.gov/mgg/global/global.html. Together, sample depth and bottom depth describe the position in the water column of each record.

Prior to analysis, we removed 14 sample depths and 1 bottom depth that were negative (most likely intertidal records). Sample depth occasionally exceeded bottom depth (for 7% records), generally by ≤20m but occasionally by >100m. We make the single reasonable assumption that in all such cases, samples were taken from the sea bed. The discrepancy then either arises from an inaccurate measurement of the depth at which the sample was taken, or due to the geographically less precise estimates of bottom depth (averaged over a grid with a resolution of 1 arc-minute, i.e. approximately 1 nautical mile at the equator) compared to the point measurements of sample depth. Because we cannot distinguish these two possibilities, in all cases we assume the sample depth is correct, and set the bottom depth equal to the sample depth. This has no qualitative bearing on our conclusions. Summing the number of records for each unique combination of bottom depth and sample depth results in a working dataset of 6987676 individual records sampled from 172012 unique locations in three-dimensional (latitude, longitude, sample depth) space. Records were drawn from depths ranging from 0—10670m, encompassing most of depth range of the global oceans.

### Data Analysis

We first plot the total number of OBIS records obtained from each distinct bottom depth to provide a global picture of the distribution of recorded marine biodiversity in regions of the ocean of differing depths. Of course, different bottom depths are not equally distributed in the marine environment, and so we next divide the global oceans into five well-recognised zones, based on bottom depth (Table 1). We then used a global sea floor topography [19] to determine the proportional area of the oceans occurring in each zone. For example, the continental shelf (<200m) accounts for around 8.7% of the area of the oceans, whereas the abyssal plain covers almost 50% of the ocean area (Table 1), and is, by surface area, the largest habitat on Earth [8]. Plotting the proportion of OBIS records originating from each zone against the proportional area of that zone shows which areas of the ocean are proportionately under- or over-represented within OBIS.

**Table 1:** Depth zones of the ocean, with the percentage of the global oceans occurring within each zone (estimated from data described in Ref. 17).

Depth	Zone	Percentage of Ocean Area	Cumulative Percentage	Depth resolution
0—200m	A. Continental Shelf	8.7	8.7	50m^a^
200—1000m	B. Continental Slope / Mesopelagic	5.8	14.5	100m
1000—4000m	C. Continental Slope / Bathypelagic	36.3	50.8	200m
4000—6000m	D. Abyssal Plain	48.6	99.4	200m
>6000m	E. Hadal Zone	0.6	100	1000m

a Surface waters to 200m deep were subdivided into 50m strata for global analyses, but into 10m strata for analyses of the Continental Shelf and Mesopelagic Continental Slope.

The letters (A-E) used for each depth zone match those used in Figures 2 and 3. Depth resolution refers to the subdivision of the water column to each depth zone used in subsequent analyses.

We next incorporate information on the position within the water column from which each record was sampled (sample depth) to create a three dimensional picture of recorded marine biodiversity. To add finer resolution to this picture, we further stratified these broad depth zones described in Table 1. Surface waters (0—200m) were divided into 50m strata, waters between 200 and 1000m deep into 100m strata, waters from 1000 to 6000m deep into 200m strata, and waters over 6000m deep into 1000m strata. So, for example, the water column above a bottom depth of 900–1000m was subdivided into 50m strata to a depth of 200m, and 100m strata thereafter. We then populated this matrix of bottom depth x sample depth with the total number of OBIS records occurring in each of the 903 cells in which sample depth ≤ bottom depth. Different cells within this matrix represent different volumes of ocean. For instance, a 50m depth interval over the abyssal plain represents more volume than the same division over the continental shelf, because the former habitat has a much larger surface area (Table 1). We therefore divided the total number of records in each cell by the relative volume of that cell, to give a standard scale throughout the water column and across all bottom depths of records per (arbitrary) unit water volume. Finally, we log_10_ transformed this standardised record number, before plotting using the *image* function in the statistical package R 2.9.2 [20]. To provide a more detailed view of shallower waters, we re-did this analysis with a finer depth resolution of 10m for water <200m deep over bottom depths over 0—1000m. The R code used for all anlyses and figures is available in Appendix S1.

The resulting figure reflects both general trends for the total number of OBIS records to vary with bottom depth, as well any trend for the distribution of sampling effort to vary through the water column. To show more clearly how the number of records varies through the water column, regardless of bottom depth, we re-calibrated the sample depth x bottom depth matrix so that each column summed to 1, allowing us to visualise separately for each bottom depth the proportion of records occurring at each sample depth. We use this to calculate for each of the five ocean zones defined in Table 1 the proportion of records from midwater. We define midwater for depths ≤200m as all records except those within 10m of the surface or of the sea bed; for depths >200m, we exclude records within 100m of the surface and within 100m, 200m or 1000m of the seabed respectively for the mesopelagic zone, bathypelagic zone and abyssal plain, and hadal zone.

## Results

**Figure 1:**
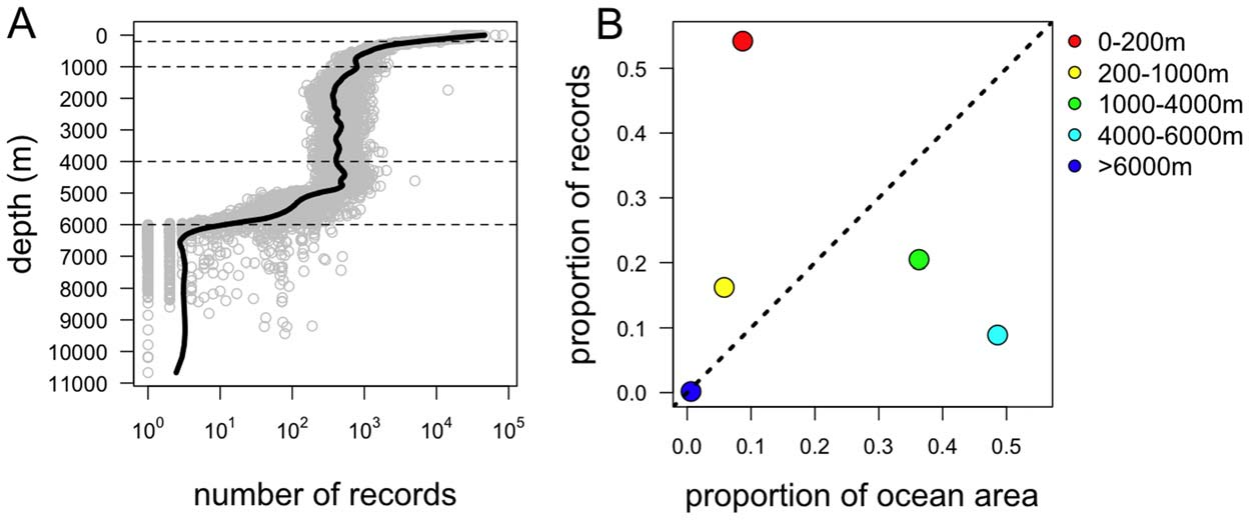
The depth distribution of OBIS records of global marine biodiversity. A. Number of OBIS records against ocean depth (grey symbols); the general trend is illustrated with a lowess smooth (solid line). Horizontal dashed lines indicate the divisions into regions A-E defined by depth at 200, 1000, 4000 and 6000m (see Table 1). B. The proportion of all OBIS records occurring in the different depth zones identified in Table 1, against the proportion of the global ocean that occurs at those depths. The 1∶1 line identifies those areas of the ocean with proportionately more (points above the line) or fewer (points below the line) records than expected given their area. This gives a conservative view of under- and over-representation based on the volume of each habitat.

The majority of our knowledge of marine biodiversity is derived from samples drawn from shallow seas: the number of records in OBIS declines precipitously with increasing bottom depth (Figure 1A). Areas of the ocean with a relatively shallow bottom (<200m) typically have thousands of associated records, whereas the deep oceans (>6000m) generally have <10 records. The lowess smooth in Figure 1A indicates that the decline in record numbers is steepest in the range 0—1000m, and again around 5000—6000m. In part this may be related to different depths of ocean having different areas. For instance, the low numbers of records in the hadal zone may be due to the fact that this zone constitutes a very small proportion of the total area of the ocean (Table 1). By plotting the proportion of all records occurring within each of the five ocean regions described in Table 1 against the proportion of ocean area that region encompasses (Figure 1B), the simultaneous effects of proportional area and number of records can be untangled. Thus, although the hadal zone has about the number of records expected, given its tiny area, other zones are either highly under-or over-represented. For instance, *>*50% of the OBIS records come from the continental shelf, which constitutes <10% of the ocean, whereas <10% of records are from the Abyssal Plain (4000—6000m), which constitutes *c*. 50% of the ocean area (Figure 1B).

**Figure 2:**
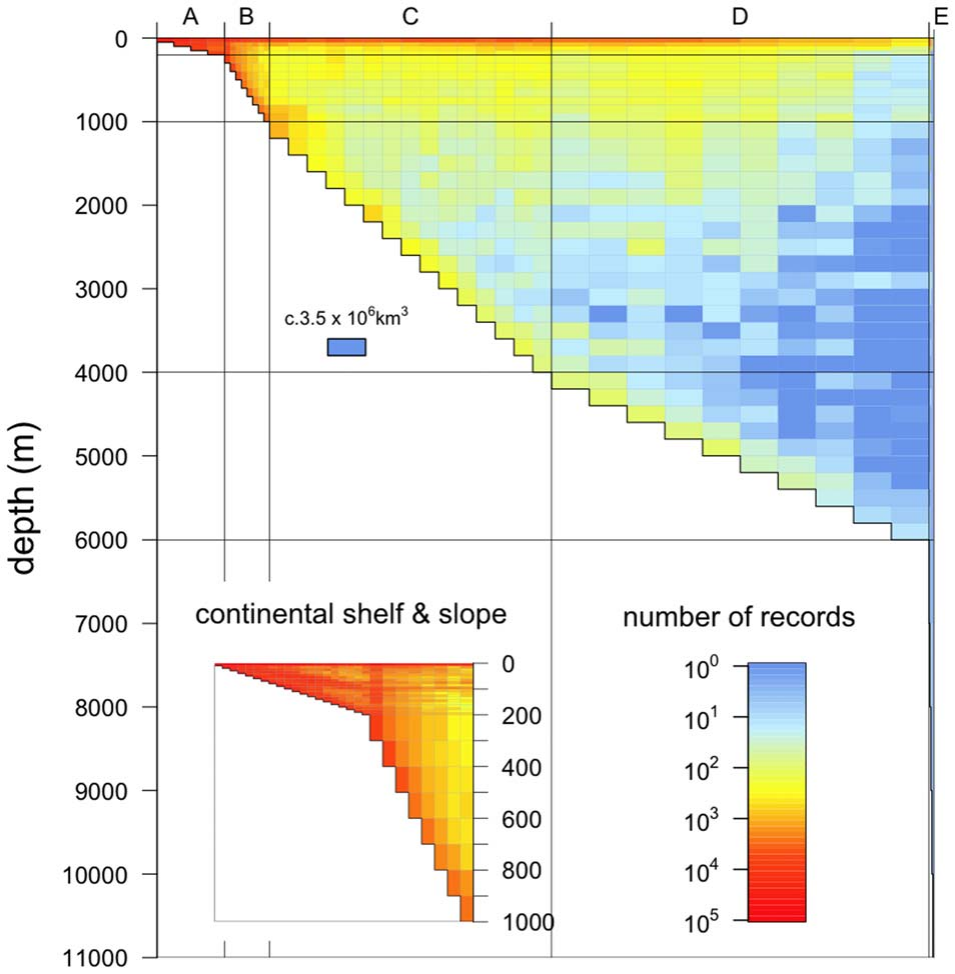
Global distribution within the water column of recorded marine biodiversity. The horizontal axis splits the oceans into five zones on the basis of depth (see Table 1), with the width of each zone on this axis proportional to its global surface area. The vertical axis is ocean depth, on a linear scale. This means that area on the graph is proportional to volume of ocean. For instance, in the deep sea each cell of 200m depth represents *c.* 3.5×10^6^ km^3^ (see cell drawn separately for scale). The number of records in each cell (each unique combination of sample and bottom depth, following the scheme in Table 1) is standardised to the volume of water represented by that cell, and then log_10_-transformed. The inset shows in greater detail the continental shelf and slope, where the majority of records are found.

The global distribution through the water column of recorded marine biodiversity is shown in Figure 2. Even on the logarithmic scale of number of records, the dominance of shallower waters within the OBIS database is clear. It is also clear, both throughout the oceans and over the continental shelf and slope, that most records have come from a narrow band of water at the ocean surface, or from the sea bed. The blue area throughout large parts of the deep pelagic ocean in particular reflects the paucity of records from this habitat. When we consider that each cell of 200m depth over the abyssal plain represents a volume of *c*. 3.5 million km^3^, the immense volume of water from which biological records are essentially absent becomes starkly apparent.

**Figure 3:**
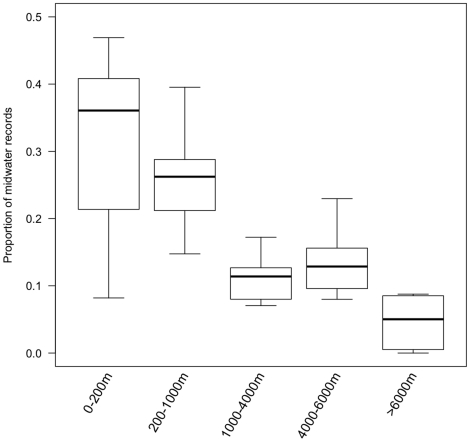
The proportion of recorded marine biodiversity originating from the midwater pelagic ecosystem. The ocean is split into the depth zones defined in Table 1. Midwater is defined as all of the water column except the 10m nearest the surface or the 10m (for the continental shelf) or 100m (for the mesopelagic continental slope) above the sea bed, and for the other ocean zones as the water column excluding the 100m nearest the surface and the 200m (for the bathypelagic zone and the abyssal plain) or 1000m (for the hadal zone) above the sea bed. For each zone, the plot shows the median, interquartile range and total range of observed proportions. The shallowest two depths (0—10m and 10—20m) are excluded, as there is no midwater according to our definition.

The general lack of pelagic records is shown more clearly by considering the proportion of records at each position in the water column separately for each bottom depth (see Figure S1). More generally, midwater diversity is under-represented at all depths: in no zone of the ocean do midwater records make up more than 50% of all records (Figure 3). However, this pattern is especially marked in the deep sea: there is a significant negative correlation between bottom depth and the proportion of midwater records (Spearman's rank correlation, *r*_s_ = -0.66, d.f. = 54, *P*<0.0001).

## Discussion

Our results clearly show that the deep oceans are vastly under-represented in OBIS, the world's largest provider of information on the distribution of marine species. More than this, however, we have also shown that midwater habitats throughout the global oceans are under-recorded relative to surface waters and the sea bed. Taken together, this shows that the least well recorded region of the marine environment is the largest by volume: the deep pelagic ocean.

There are two possible explanations for this: either the deep pelagic ocean is especially low in biomass, or it has been especially under-sampled (or some combination of the two). Historically, the first of these possibilities has been espoused. For instance, Charles Wyville Thomson, leader of the Challenger Expedition in the 1870s which effectively launched the discipline of deep sea biology [11], believed that ’the fauna of deep water is confined primarily to two belts, one at and near the surface and the other on and near the bottom; leaving an intermediate zone [i.e., the deep pelagic] in which larger animals… are nearly or entirely absent' (quoted in Ref. 9). More recent evidence suggests, however, that it is under sampling and net avoidance rather than a lack of organisms that generate the patterns we have observed.

However, new technologies have dramatically altered perceptions of the deep pelagic ecosystem [10], [21], suggesting that with past techniques, even high sampling effort may not have resulted in correspondingly high numbers of biological specimens being collected. The ability to view animals *in situ* means that the diversity of organisms not captured by traditional sampling methods, such as the gelatinous fauna that constitutes up to a quarter of pelagic biomass [10] is now better understood. Importantly, their abundance is now known to be much higher than most deep-sea biologists expected [10]. For instance, the recently discovered new clade of large, active deep sea annelids (including holopelagic species) occur at high biomass [12]. Thus, the deep pelagic appears to conform to dictum that the more you survey, the more you find, as witnessed recently in other marine habitats including fish in the hadal zone [22] and microbes in surface waters [23], [24]. Such findings have led to estimates of a million undescribed species in the deep pelagic [10] and the proposal that ‘within this vast midwater habitat are the planet’s largest animal communities… These animals probably outnumber all others on Earth' (Ref. 10∶848). Clearly, there is much work still to be done before we can draw conclusions regarding the depth distribution of *actual* marine biodiversity from databases of *recorded* marine biodiversity.

Increasing our understanding of these communities is important for a number of reasons. First are the ecosystem services they provide, for instance supporting global fisheries, climate regulation, and bioprospecting [11]. In addition, they have considerable potential as a model system for testing biogeographic hypotheses, such as large-scale gradients in diversity. The deep pelagic environment is spatially homogeneous and has been very stable over time, with little in the way of seasonal and latitudinal variability [10], and yet latitudinal gradients appear to exist in the diversity of at least some deep pelagic taxa [8]. Might this provide a means to tease apart the confounding effects of the environment, geometric constraints, and species tolerances in explaining biogeographic patterns [25], [26]? More generally, it may prove easier to unravel the multiple drivers of change in marine ecosystems, including historical human influences and future climate change, by studying those habitats that have been least affected to date – the mid-ocean, mid-water environment – before transferring this understanding back into more heavily disturbed coastal and benthic systems [27].

Finally, even if pelagic ecosystems remain less impacted than coastal regions, there is increasing concern that human activities including fishing, pollution and climate change have already had substantial effects, and that these pressures are only likely to increase in future [11], [28]-[30]. Although some conservation measures, in particular the establishment of pelagic marine protected areas, may be possible in the absence of detailed biological information [28], clearly an increased understanding of the temporal and spatial dynamics of pelagic organisms will improve their effectiveness. We hope that exposing biodiversity's big wet secret will stimulate further exploration of Earth's biggest ecosystem.

## Supporting Information

Appendix S1 R code used for data processing and production of all figures.
(0.02 MB TXT)

Figure S1 Global distribution of recorded marine biodiversity expressed as the proportion of OBIS records occurring at each position in the water column over each bottom depth. Only those cells (unique combinations of sample and bottom depth, following the scheme in Table 1) contributing more than 1% of records from a given bottom depth are coloured. To provide an alternative scale to Figure 2, where the size of each cell represents the volume of water it contains, here we transform both the vertical depth axis (d) and the horizontal area axis (a) (d′ = d^2/3^, a′ = √a). This better shows the relatively high number of records in surface waters, as well as patterns in the smaller depth zones (e.g., continental shelf, hadal zone).
(0.17 MB PDF)
