# Supplementary figures and images for: Biodiversity's Big Wet Secret: The Global Distribution of Marine Biological Records Reveals Chronic Under-Exploration of the Deep Pelagic Ocean
Source: PLoS One. 2010 Aug 2;5(8):e10223. doi: 10.1371/journal.pone.0010223 (PMC2914017; doi:10.1371/journal.pone.0010223)

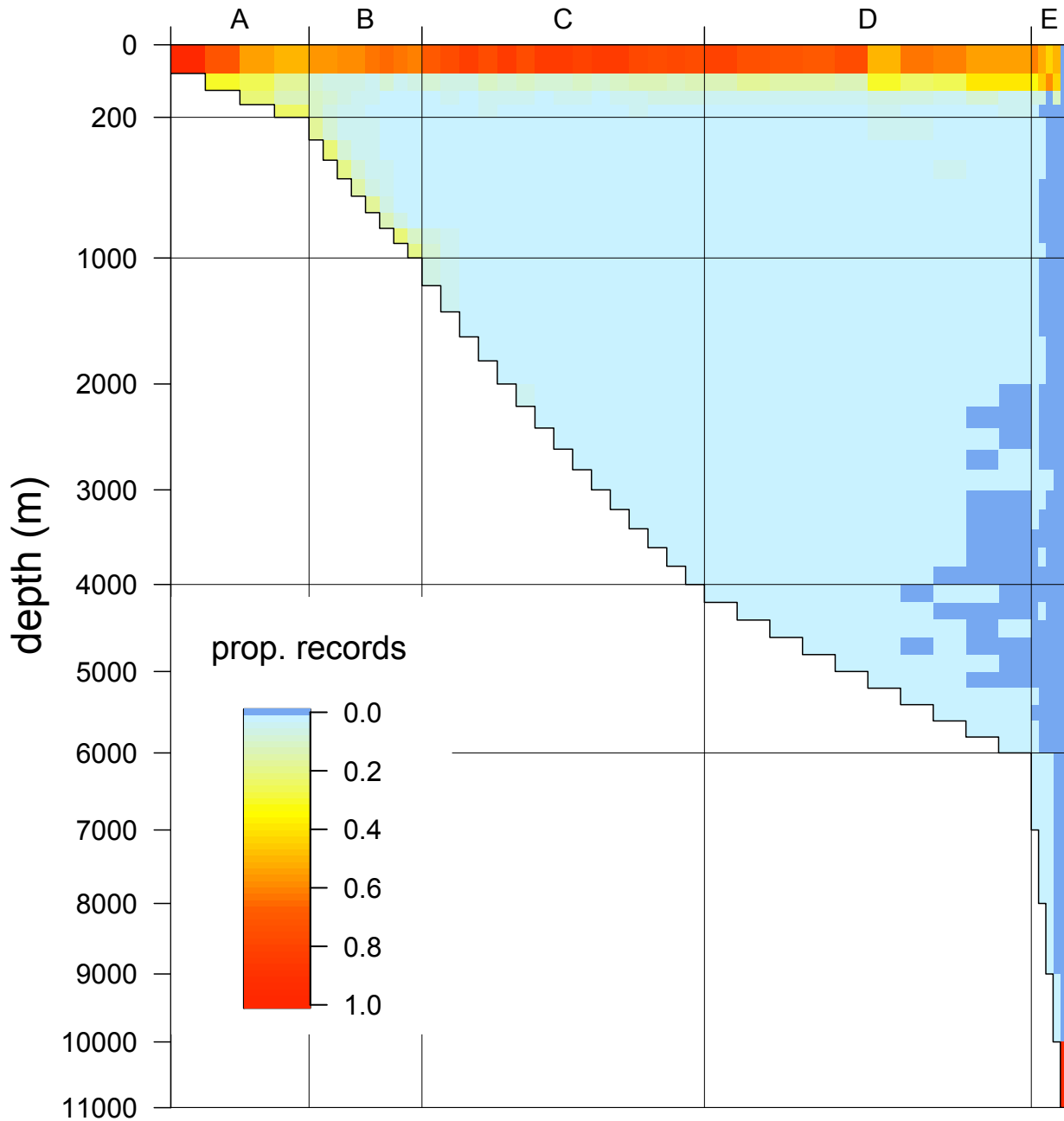

Supplement: Figure S1 — Global distribution of recorded marine biodiversity expressed as the proportion of OBIS records occurring at each position in the water column over each bottom depth. Only those cells (unique combinations of sample and bottom depth, following the scheme in Table 1) contributing more than 1% of records from a given bottom depth are coloured. To provide an alternative scale to Figure 2, where the size of each cell represents the volume of water it contains, here we transform both the vertical depth axis (d) and the horizontal area axis (a) (d′ = d2/3, a′ = √a). This better shows the relatively high number of records in surface waters, as well as patterns in the smaller depth zones (e.g., continental shelf, hadal zone). (0.17 MB PDF) [file pone.0010223.s002.pdf]
